# Recent physical conditions and health service utilization in people with common mental disorders and severe mental illness in England: Comparative cross-sectional data from a nationally representative sample

**DOI:** 10.1192/j.eurpsy.2020.22

**Published:** 2020-02-20

**Authors:** Kurt Buhagiar, Georgia Templeton, David P. J. Osborn

**Affiliations:** 1 Department of Research, Innovation & Medical Education, East London NHS Foundation Trust, London, United Kingdom; 2 Division of Psychiatry, University College London, London, United Kingdom

**Keywords:** common mental disorders, comparative study, healthcare utilization, physical health, severe mental illness

## Abstract

**Background.:**

Policies addressing the physical health of people with mental disorders have historically focused on those with severe mental illness (SMI), giving less prominence to the more prevalent common mental disorders (CMDs). Little is known about the comparative physical health outcomes of these patient groups. We aimed to first compare the: (a) number of past-year chronic physical conditions and (b) recent physical health service utilization between CMDs vs. SMI, and secondly compare these outcomes between people with CMDs vs. people without mental disorders.

**Methods.:**

We analyzed cross-sectional data from the third Adult Psychiatric Morbidity Survey, a representative sample of the English population. We determined the presence of physical conditions and health service utilization by self-report and performed logistic regression models to examine associations of these outcomes between participant groups.

**Results.:**

Past-year physical conditions were reported by the majority of participants (CMDs, *n* = 815, 62.1%; SMI = 27, 63.1%) with no variation in the adjusted odds of at least one physical condition between diagnoses (odds ratio [OR] = 0.96, 95% confidence intervals [CI] 0.42–1.98, *p* = 0.784). People with CMDs were significantly more likely to be recently hospitalized relative to with those with SMI (OR = 6.33, 95% CI 5.50–9.01, *p* < 0.05). Having a CMD was associated with significantly higher odds of past-year physical conditions and recent health service utilization (all *p* < 0.001) compared with the general population.

**Conclusions.:**

People with CMDs experience excess physical health morbidities in a similar pattern to those found among people with SMI, while their somatic hospitalization rates are even more elevated. Findings highlight the importance of recalibrating existing public health strategies to bring equity to the physical health needs of this patient group.

## Introduction

The association between poor physical health and severe mental illness (SMI), traditionally defined as schizophrenia-like disorders, bipolar disorder, and other nonorganic psychoses, is now well-established and has gained prominent research and policy attention over the years [1].

People with SMI are at particular risk for increased physical health morbidity, given their higher risk of unhealthy lifestyle choices such as smoking [2,3], treatment with antipsychotic drugs [4], social deprivation [5], diminished awareness of their poor physical health [2,6], and delayed treatment for their physical problems [7] relative to the general population. The combination of these risk factors leads to excess disability, culminating in reduced life-expectancy [8], and excess mortality [9] that have been increasing over the years relative to the general population [10,11]. In light of these serious physical health risks, public health policies, and treatment interventions have therefore focused their attention on people with SMI [12], as opposed to other patient groups with more prevalent common mental disorders (CMDs) such as anxiety disorders and unipolar depression.

Despite being mostly treated in primary care, people with CMDs also have a very complex association with physical disorders acting in a multifaceted and bidirectional pathway that increases disability and health resources [13,14]. Previous studies have consistently demonstrated elevated risks of physical comorbidity among people with CMDs compared with the general population [15,16], predisposing to even worse outcomes than having either illness alone [13,17]. Additionally, the prevalence of unhealthy lifestyle behaviors among people with CMDs may be comparable to those observed in people with SMI in some instances [18]. SMI and CMDs as groups of disorders, however, differ markedly from one another from both psychological and sociodemographic perspectives [19], contributing to variations in their progression, which may subsequently also influence comparative physical health needs and outcomes [20,21].

Despite the wealth of research evaluating physical comorbidities in people with SMI, studies at population-based level have not generally explored comparative associations relative to people with CMDs [17]. The physical health needs of people with CMDs compared with those of people with SMI have therefore not been fully quantified and appear to be less well understood [12]. In addition, there are no UK-specific data on the relationship of CMDs and physical conditions relative to general population, which may be affected by unique geographical factors and intricacies of healthcare systems. The prevalence of CMDs globally is about 10-fold higher than that of SMI [22], hence shows a much greater impact on overall morbidity at population-based level. However, to date, there exist no policies and treatment interventions aimed specifically at people with both CMDs and physical conditions, in contrast with the extensive attention given internationally to people with SMI with similar physical health problems [12].

We need to understand whether there are quantitative differences in the physical health outcomes between these two distinct patient groups. In turn, this would clarify whether there is a need to recalibrate public health policy to make equitable focus on people with all types of chronic mental disorders and not merely SMI.

The primary aim of the current study was to compare (a) the prevalence of recent physical conditions and (b) recent health service utilization for physical conditions among people with CMDs compared to those with SMI. Our secondary aim was to compare these associations for people with CMDs relative to those without mental illness. We hypothesized that people with CMDs have: (a) comparable physical health outcomes and utilization relative to people with SMI and (b) higher risk of physical conditions and health service utilization compared with people without mental illness.

## Methods

### Sampling and interviewing procedures

We conducted a secondary analysis of data from the Adult Psychiatric Morbidity Survey 2007 (APMS 2007) [23], a sample survey of all adults living in private households in England, collecting nationally representative cross-sectional data for the population aged 16 and above. The dataset has been made publicly available via the U.K. Data Service [24], while the full sampling methodology is available in the main survey report published elsewhere [23]. In summary, the survey adopted a multistage stratified probability sampling design, according to socio economic grouping and geographical region, yielding a total of 7,461 actual participants (response rate = 57%), with eventual usable data from 7,403 participants. Data were weighted to take account of nonresponse and reduce selection bias so that the results were representative of the entire population (see Supplementary Note 1). Ethical approval was obtained from the Royal Free Hospital and Medical School Research Ethics Committee (ref 06/Q0501/71).

### Main measures

#### Psychiatric morbidity

*Common mental disorders:* The Clinical Interview Schedule Revised (CIS-R) [25] was used to identify 14 categories of nonpsychotic symptoms. The CIS-R has two initial filter questions for each of these categories of symptoms covering 1 month prior to the interview, with a positive answer leading to more detailed enquiry about symptoms in the previous week. Responses were then entered into a computer algorithm to subsequently generate six categories of CMDs in the previous week according to ICD-10 criteria: depression, generalized anxiety disorder, panic disorder, phobia, obsessive–compulsive disorder, and mixed anxiety and depressive disorder. For our analyses, we used a derived variable combining the prevalence estimates of all six diagnostic categories.

*Severe mental illness:* People with probable psychotic disorder were identified if they responded positively to one of several screening questions in the survey, as detailed in the APMS report [23] (see Supplementary Note 2). If they did, participants were then eligible for a further clinical assessment for a diagnosis of definitive psychotic disorder using the Schedule for Clinical Assessment in Neuropsychiatry version 2.1 (SCAN) [26]. For our analysis, we assigned participants meeting criteria for either probable or definitive psychotic disorder in the past year to a single group as a proxy for SMI, consistent with previous research [27].

*People without mental disorders:* We identified a group of participants who did not meet the diagnostic criteria for any of the mental disorders assessed in the survey, including personality disorders, dependence on alcohol or drugs, history of self-harm, and problem gambling. Hazardous use of alcohol and nonproblematic drug use were not exclusion criteria.

#### Physical comorbidity

Physical health conditions were assessed by asking participants if they had suffered from a number of mostly long-term physical conditions presented to them on a list, first in the year preceding the interview and second, in their lifetime since the age of 16 years. We included diabetes, hypertension, angina/myocardial infarction, any cancer, ear disorders/hearing problems, cataracts/eyesight problems, emphysema/bronchitis, asthma, bladder problems/incontinence, bowel/colon problems, stomach ulcers/digestive problems, musculoskeletal problems, infectious diseases, and skin problems. We specifically excluded epilepsy, dementia, and migraine due to their potential symptomatic overlap with mental disorders. We then generated a categorical variable for past-year physical conditions: (a) having at least one physical condition or not and (b) the total number of physical conditions reported per participant, classifying participants into four groups around the median consistent with previous studies [28].

#### Health service utilization and treatment

*Physical health service utilization:* Participants were asked about their recent use of health services for physical health problems with respect to (a) consultations in person or by telephone with their general practitioner (GP) in the past year; (b) planned visit to an out-patient clinic in the previous 3 months; and (c) acute admission to a general hospital in the previous 3 months, defined as at least one overnight stay except for childbirth.

*Mental health treatment:* Data regarding named psychotropic medications and mental health service use received by participants were collected, the latter acting as a proxy for receipt of secondary psychiatric care.

#### Covariates

*Socio demographic variables:* age, gender, ethnicity, marital status, educational qualifications, occupation, and debt.

*Clinical variables:* We generated variables for body mass index (BMI; obesity, BMI ≥ 30 kg/m^2^) and current smoking status. The Alcohol Use Disorders Identification Test (AUDIT) [29] was used as a screening tool for patterns of harmful and hazardous drinking, followed by the Severity of Alcohol Dependence Questionnaire (SAD-Q) [30] to determine dependence. We created a dichotomous variable for participants with a drinking problem (hazardous use or dependence) vs. those without a drinking problem during the past 6 months. Participants were also asked about their drug intake using a computer-assisted self-completion interview followed by a selection of questions from the Diagnostic Interview Schedule [31] for each drug reported. We used a dichotomous variable, classifying participants into being dependent on any drug or not.

### Data analysis

Data analysis was conducted using the *survey* commands in Stata 12.0 for Macintosh (Stata Corp LP, College Station, TX). These commands allow for the use of clustered data inherent in complex survey designs modified by probability weights and provide robust estimates of variance. The weighting procedure is necessary in order to account for potential biases that may be caused by nonresponse.

We described variables using actual counts, but proportions and ratios are derived from weighted measures. We first compared sociodemographic and clinical variables between the CMD and SMI groups, using Pearson chi-square tests with Rao and Scott second-order correction for survey data for categorical variables or Student’s *t* tests for continuous variables. First, we calculated the total number of past-year physical conditions in each participant group. We then used univariable and multivariable logistic regression analysis to compare associations across people with CMDs and SMI, the latter acting as a reference group. The multivariate models were conducted in accordance with previously published studies addressing similar research questions [32] and included potentially confounding sociodemographic covariates associated with both mental disorders and physical health outcomes: age, gender, ethnicity, employment, education, as well as covariates for behavior risk factors: BMI, smoking status, alcohol intake, and drug dependence. Although the latter group of covariates may be regarded as potential mediators of associations between mental disorders and health outcomes, they themselves also represent directly or indirectly psychiatric comorbidities, hence also acting as potential confounders in these associations of interest [32].

Second, we used univariable logistic regression in order to compare the odds of physical health service utilization (GP visits, outpatient visits, and acute somatic hospitalization) between the two main groups of interest, followed by a multivariable logistic regression analysis adjusting for the number of reported lifetime physical disorders in addition to the selected covariates above.

A second set of analyses was repeated for all of these physical health outcomes, comparing people with CMDs with participants without mental disorders.

## Results

### Sample characteristics

CMDs were identified in 1,248 participants, meeting the criteria for the following specific ICD-10 diagnoses: severe depression (*n* = 85, 6.3%), moderate depression (*n* = 63, 4.8%), mild depression (*n* = 88, 6.2%), panic disorder (*n* = 67, 6.0%), obsessive–compulsive disorder (*n* = 31, 2.8%), social phobia (*n* = 37, 2.7%), agoraphobia (*n* = 16, 1.3%), specific phobia (*n* = 19, 1.7%), mixed anxiety and depression disorder (*n* = 637, 52.4%), and generalized anxiety disorder (*n* = 207, 15.7%). There were 40 participants with SMI and no comorbid CMDs, while 5,695 participants did not meet the criteria for any mental disorder. Table 1 summarizes the sociodemographic and clinical characteristics of the two main participant groups. Participants with CMDs were significantly less likely than those with SMI to have been in contact with secondary care services for mental disorders (Table S1).

**Table 1.** Characteristics of participants with common mental disorders and severe mental illness

Variable	CMDs^a^ (*n* = 1,248)		SMI^a^ *(n = 40)*		*χ^2b^ (df)*	*p*
	*n*	%	*n*	%		
Gender, female	835	62.5	27	59.1	0.16 (1)	0.696
Age band (years)						
16–34	323	34.7	10	27.7	3.81 (3)	0.206
35–54	513	39.9	22	55.5		
55–74	319	19.9	7	15.3		
75+	93	5.4	1	1.5		
Ethnicity						
White	1115	88.5	34	92.0	0.38 (1)	0.532
Black or minority^c^	113	11.5	3	8.0		
Marital status						
Married or cohabiting	567	54.4	14	48.9	0.47 (2)	0.751
Single	307	26.8	14	31.6		
Widowed, separated, or divorced	374	18.9	12	19.5		
Educational qualifications						
Further or higher education^d^	249	22.0	6	17.0	0.47 (1)	0.520
Secondary education only	961	78.0	32	83.0		
Employment status						
Employed	581	52.3	7	30.5	15.84 (2)	<0.001
Unemployed	667	47.8	33	59.5		
Currently in debt	986	79.2	26	67.6	3.23 (1)	0.303
Obese^e^	279	22.7	7	19.6	0.36 (1)	0.596
Current smoking	427	34.6	21	49.1	3.19 (1)	0.260
Problem drinking^f^	339	30.0	10	26.9	0.16 (1)	0.937
Drug dependence^g^	77	7.8	7	17.1	3.65 (1)	0.049

Abbreviations: CMD, common mental disorders; SMI, severe mental illness.

a Percentages are weighted to account for complex survey design. Counts may not add up to totals due to missing data.

b Pearson’s *χ* ^2^ with Rao and Scott second-order correction for survey data analysis.

c Includes South Asian and mixed race participants.

d Includes university degrees or professional qualifications only.

e BMI ≥ 30 kg/m^2^.

f In the past 6 months, assessed with the Severity of Alcohol Dependence Questionnaire.

g In the past year, assessed with the Diagnostic Interview Schedule.

### Comparison between CMDs and SMI

#### Physical disorders

Nearly one-third of all participants reported having at least one past-year physical condition (CMDs, *n* = 815, 62.1%; SMI = 27, 63.1%), with no statistically significant difference between participant groups in both unadjusted (odds ratio [OR] = 0.93, 95% confidence intervals [CI] 0.47–1.79, *p* = 0.873) and adjusted models (OR = 0.96, 95% CI 0.42–1.98, *p* = 0.784). Repeating the analysis using categorical variables according to the total number of self-reported past-year physical conditions also failed to reveal variation between the two groups, even when confounders were taken into account (Table 2).

**Table 2.** Physical conditions in the past year among people with common mental disorders compared with people with (a) severe mental illness and (b) without mental disorders: weighted self-reported prevalence and results of logistic regressions for their association with participant groups

Number of past-year physical disorders	CMDs^a^ (*n* = 1,248)		SMI^a^ (*n* = 40)		No mental disorders^a^ (*n* = 5,695)		Unadjusted OR (CMDs vs. SMI) (95% CI)		Adjusted OR^b^ (CMDs vs. SMI) (95% CI)		Adjusted OR^b^ (CMDs vs. no mental disorders) (95% CI)	
	*n*	(%)	*n*	(%)	*n*	(%)						
None	433	(37.9)	13	(36.9)	2058	(42.1)	–		–		–	
1	389	(32.4)	14	(32.8)	1646	(28.8)	0.96	(0.42–2.22)	1.03	(0.43–2.99)	1.71	(1.41–2.08)^**^
2–3	328	(22.9)	10	(25.9)	1520	(22.9)	0.86	(0.35–2.08)	1.01	(0.38–2.63)	3.21	(2.63–3.94)^**^
≥4	98	(6.8)	3	(4.4)	473	(6.2)	1.49	(0.41–5.56)	1.89	(0.48–7.69)	7.35	(5.60–9.66)^**^

Abbreviations: CI, confidence interval; CMD, common mental disorders; OR, odds ratio; SMI, severe mental illness.

a Percentages are weighted to account for complex survey design.

b Adjusted for age, body mass index, gender, ethnicity, education, employment, smoking, alcohol consumption, and drug dependence.

** *p* value <0.001.

#### Health service utilization

As summarized in Table 3, people with CMDs had significantly elevated odds of acute hospital admissions for physical disorders in the past 3 months even after adjusting for participant characteristics and number of lifetime physical conditions (OR = 6.33, 95% CI 5.05–10.0, *p* = 0.045), despite similar rates of GP and out-patient visits.

**Table 3.** Physical health service utilization among people with common mental disorders relative to people with (a) severe mental illness and (b) people without mental disorders

Physical health service utilization	CMDs^a^ (*n* = 1,248)		SMI^a^ (*n* = 40)		No mental disorders^a^ (*n* = 5,695)		Adjusted OR^b^ (CMDs vs. SMI) (95% CI)		Adjusted OR^b^ (CMDs vs. no mental disorders) (95% CI)	
	*n*	(%)	*n*	(%)	*n*	(%)				
Spoke to GP in past year^c^	939	(71.9)	32	(77.3)	3,588	(60.4)	0.86	(0.37–1.96)	1.82	(1.55–2.14)^**^
Attended out-patient clinic^d^	371	(26.8)	9	(20.8)	1,092	(17.9)	1.45	(0.60–3.57)	1.84	(1.57–2.17)^**^
Admitted to hospital^d^	75	(5.18)	1	(1.7)	171	(2.54)	6.33	(5.05–10.0)^*^	2.29	(1.67–3.14)^**^

Abbreviations: CI, confidence interval; CMDs, common mental disorders; GP, general practitioner; OR, odds ratio; SMI, severe mental illness.

a Percentages are weighted to account for complex survey design.

b Adjusted for age, body mass index, gender, ethnicity, education, employment, number of physical disorders, smoking, alcohol consumption, and drug misuse.

c In person or by telephone regarding a physical health problem.

d For physical health problem only except for giving birth in previous 4 months.

* *p* value <0.05.

** *p* value <0.001.

#### Supplementary analyses

Comparing a subgroup of participants with CMDs who had consulted their GP in the past year for a mental health problem (*n* = 512, 41.3%) relative to the total SMI group, revealed findings consistent with those of the total CMD sample (past-year physical conditions, adjusted OR = 0.89, 95% CI 0.46–1.81, *p* = 0.797; somatic GP consultations, adjusted OR = 0.85, 95% CI 0.32–1.97, *p* = 0.598; somatic out-patient visits, adjusted OR = 1.38, 95% CI 0.56–3.76, *p* = 0.562; and somatic hospital admissions, adjusted OR = 5.21, 95% CI 4.13–8.7, *p* = 0.036). The smaller subgroup of CMD participants already taking psychotropic medications or undergoing psychotherapy at the time of the interview (*n* = 317, 24.2%) also reported similar rates of physical conditions in the past year relative to the SMI participants (adjusted OR = 0.91, 95% CI 0.32–1.86, *p* = 0.626).

To further test the robustness of our findings, we finally compared people with SMI with people without mental illness with respect to all outcomes of interest. Despite the elevated odds among people with SMI to report at least one past-year physical condition (OR = 3.46, 95% CI 1.23–8.04, *p* = 0.038), their odds for acute somatic hospitalization were nearly half as much (OR = 0.54, 95% CI 0.07–0.83, *p* = 0.032). Full results of these additional analyses are summarized in Table S2.

### CMDs vs. people without mental illness

#### Physical disorders

People with CMDs had significantly higher odds of reporting physical conditions in the past year (OR = 1.21, 95% CI 0.98–2.26, *p* < 0.001; adjusted OR = 1.82, 95% CI 0.96–2.16, *p* < 0.001) compared with people without mental illness. As shown in Table 2, this trend continued when physical disorders were also analyzed as categorical variables, with the odds for greater multimorbidity among people with CMDs becoming increasingly elevated.

#### Health service utilization

People with CMDs were significantly more likely than the general population to seek medical help for their physical conditions in both primary and secondary care (Table 3).

## Discussion

In this population-based study of a nationally representative study in England, we found that nearly two-thirds of participants with either CMDs or SMI reported having at least one physical condition in the past year. In addition, we found no statistically significant variation between people with CMDs and those with SMI in the odds of having past-year physical conditions, even after taking into account socio demographic variables and modifiable risk factors predisposing to poor physical health. Although people with CMDs and SMI visit their GP or outpatients for physical health problems just as often, people with CMDs are considerably more likely to be recently hospitalized for physical complaints even when physical multimorbidity was controlled for. Direct comparisons across distinct groups of people with mental illness, as opposed to comparisons with the general population, have seldom been explored before.

Through our secondary objectives, we confirmed the elevated odds of physical conditions and physical health service utilization in people with CMDs compared with the general population as seen in international literature [15,16]. The odds of severe past-year physical health multimorbidity (≥4 conditions) among people with CMDs were over seven times as elevated.

### Interpretation of findings and comparison with other studies

Only a very small proportion of people with CMDs (c. 3%) in our sample had received specialist psychiatric care, implying less severe symptomatology overall, yet their physical health compared with that of the general population was poor and even comparable with that of people with SMI. The chronic nature and the multiple past-year physical conditions present in a large proportion of people with CMDs are also indicative of the high rates of active physical multimorbidity, in line with previous findings elsewhere [15–17].

Given the complex bidirectional relationship between CMDs and physical disorders, within the context of the study’s cross-sectional design, the temporal relationship between the two cannot be entirely elucidated. In other words, it is possible that a proportion of individuals meeting diagnostic criteria for CMDs at the time of participation might have developed neurotic symptoms as an aftermath to a recently diagnosed physical condition, rather than the CMD itself being a precursor. This may have inflated our findings with respect to the high rates of physical conditions in people with CMDs, consequently equaling those found in people with SMI. However, our sensitivity analyses exploring somatic outcomes among subgroups of CMD participants (i.e. those who made contact with their GP about mental health problems in the previous year or had already been taking treatment for a CMD at interview), revealed similar results with those of the main CMD vs. SMI analysis. Given the time-frames for inclusion in these subgroups, these participants are likely to have had at least emerging neurotic symptoms predating the onset of the physical conditions or the need for somatic hospitalization. These findings, may therefore underline the strong antecedent role of CMDs in predisposing to the later emergence of physical conditions. Recent findings from population-based longitudinal data in the United States in fact, do show a strong independent predictive effect of CMDs on a wide range of physical symptoms and diagnosable conditions, and that CMDs themselves may act as even stronger risk factors than obesity and smoking for physical conditions [33].

The comparable rates of recent physical conditions among people with CMDs relative to those with SMI are surprising *prima facie* given the public health focus on the latter group [12]. However, our findings also showed the equally elevated rates of unhealthy lifestyle risk factors (e.g., obesity, alcohol consumption, and smoking) in people with CMDs, mirroring emerging findings [18]. The lack of physical health policies specific to people with CMDs may therefore not be a reflection of their lack of true physical health needs, but perhaps the result of an inadvertent oversight by policymakers to date.

To the best of our knowledge, only one previous smaller study (total *n* = 200) based in the United States has made direct comparisons of the rates of self-reported physical conditions between people with depression and those with schizophrenia receiving community care [34], reporting findings in line with the current study. Data regarding physical conditions in both the latter and the current study were entirely obtained by self-report, calling reliability and validity into question, although studies elsewhere have evidenced the reliability of self-reports about physical conditions in people with mental illness [35,36].

The excess physical health service utilization by people with CMDs compared with the general population is well recognized [37] and replicated by our findings. People with CMDs also had markedly higher odds than people with SMI for somatic hospitalization in the preceding 4 months despite no variation in the rates of recent GP consultations and somatic out-patient visits. These excess rates of hospitalization may reflect the systematically poorer healthcare for people with SMI in hospitals [20,21,38], while paradoxically favoring people with CMDs [20,39,40]. It is well known that nonpsychiatric clinicians are less likely to make accurate somatic diagnoses and may provide suboptimal somatic health care to people with SMI relative to those with nonpsychotic mental disorders and the general population [40]. People with SMI may also be less capable to describe their physical symptoms [40], despite their motivation to access physical healthcare as frequently as those with CMDs as shown by our results. The combination of these factors may then lead to fewer hospitalizations being offered to them despite the clinical indication [40]. On the other hand, people with CMDs may be prone to describe physical symptoms more emphatically by virtue of their neuroticism, leading to more frequent admissions even in the absence of clear need [6,20]. While the differential misclassification bias resulting from these opposing factors might have inflated the true estimate of the association between somatic hospitalization and CMDs, it is unlikely that it accounts entirely for the more than sixfold higher odds identified in our study. This finding thus underlines: (a) the significant synergy between physical health problems and CMDs, acting in a complex multifaceted manner; (b) the strain exerted on inpatient physical healthcare systems by people with CMDs; and therefore (c) the specific need to prevent and improve the care of physical health problems of people with CMDs. Our findings are closely in line with those of a robust large national study in Australia, showing lower rates of somatic hospitalization for people with SMI and higher rates for people with CMDs relative to the general population [20]. A more recent study using a large dataset from a county in the east of England, also confirmed the increased risk of somatic hospitalization for people with co-occurring anxiety and depression compared to those with no mental illness, at least in those over the age of 40 [32].

### Limitations

One limitation of the study is the small size of the SMI group, indicative of the relative rarity of psychotic symptoms across the population, yet potentially undermining the statistical power of our analyses. However, both the selection methods and the weighting procedures in the survey ensured that nonresponse bias was minimized on a range of sociodemographic and area characteristics, as demonstrated by the stable prevalence of psychosis compared with the earlier versions of the survey in 1993 and 2000 [24]. Furthermore, previous studies based on these participants with SMI have been robustly reported elsewhere [27,41,42]. In one such study [41], multiple analyses were conducted to confirm the statistical power yielded by this group, as well as sensitivity analyses to confirm the validity of the results so obtained. Repeating similar power calculations and supplementary analyses for this sample was therefore beyond the scope of the current study.

Our participants with SMI do not necessarily meet the full criteria for a psychotic disorder such as schizophrenia. However, a previous analysis has demonstrated that participants reporting as little as one psychotic symptom on the PSQ share similar sociodemographic and clinical correlates to those with SMI proper [43]. Similarly, we also know that subtypes of CMDs carry heterogeneous patterns and strengths of associations with physical disorders [15], yet we did not draw a distinction in our analysis between these different subtypes. However, there are strong overlaps in the psychological constitution of CMD participants [44].

Additionally, we make tentative inferences about the relative timing of onset of physical disorders from a cross-sectional survey. The issue of bidirectionality of the association between mental and physical health outcomes is an area of great complexity [13,14], yet the design of our study precluded us from making temporal inferences with accuracy. A related issue is that of the discrepancy in the time-frame of the diagnosis (SMI, 1 year; CMD, at least 1 month). However, our sensitivity analyses that included only CMD participants with potential psychiatric symptoms in the previous year and/or already receiving psychiatric treatment, made little difference to the results.

Many of our physical conditions and physical health utilization variables were broad in their definition, although, this is an inherent limitation of using existing datasets.

The main advantage of the current study is the population-based approach. The sampling procedure ensured that a large probability sample representative of the entire population of England could be analyzed. The accompanying wealth of data provided the ability to control for important confounding covariates. Although the response rate of 57% was relatively modest, robust weighting procedures were incorporated in the analysis. The population-based approach also meant that sampling biases otherwise resulting from more circumscribed population groups employed in previous studies could be minimized. This was particularly the case in the CMD group, where only a small fraction of the participants had had contact with psychiatric secondary care. Well-validated instruments, including the CIS-R and the SCAN were used in order to ascertain the diagnosis. The use of a general population comparison group without psychiatric morbidity is a further strength.

### Clinical implications

A main objective of the UK government’s mental health policy is to improve the physical health and reduced associated mortality in people with all mental disorders [45] and a drive to push forward the integration between physical and mental health care [46]. However, policy documents focusing on the physical health of people with mental disorders have generally only made fleeting reference to CMDs [44]. A number of strategies have also been implemented aimed at reducing the gap in physical health outcomes and mortality between people with SMI and the general population [47] but no comparable recommendations exist for people with CMDs [12]. Despite these efforts, the physical health and mortality gap between people with SMI and those without mental illness continues to prevail [11]. Given this context and the findings from our study, it is safe to postulate therefore, that in the absence of similar recommendations for people with CMDs, the physical health needs in this patient group continue to be unmet. Our findings affirm the continued burden of somatic comorbidity in people with mental disorders irrespective of the SMI/CMD dichotomy. Efforts to improve physical health outcomes therefore need to be directed to people with CMDs as well as SMI.

## Conclusion

Our findings demonstrate the widespread physical health burden among people with CMDs relative to the general population, but more significantly also indicate that the physical health of people with CMDs may be just as poor as that of people with SMI. In clinical practice, a more proactive approach by policymakers and healthcare providers could ensure equity of healthcare provision and physical health outcomes across all vulnerable groups of people with mental illness.

## Data Availability

The data that support the findings of this study are openly available in the UK Data Service archive at http://doi.org/10.5255/UKDA-SN-6379-1 [24].

## Supplementary material

For supplementary material accompanying this paper visit https://doi.org/10.1192/j.eurpsy.2020.22. click here to view supplementary material
