# Supplementary material for: Recent physical conditions and health service utilization in people with common mental disorders and severe mental illness in England: Comparative cross-sectional data from a nationally representative sample
Source: Eur Psychiatry. 2020 Feb 20;63(1):e19. doi: 10.1192/j.eurpsy.2020.22 (PMC7315885; doi:10.1192/j.eurpsy.2020.22)
Supplement: Supplementary file 1 [file S092493382000022Xsup001.docx]

**Supplementary Material**

**Supplementary note 1 – Weighting of data to adjust for selection and non-response bias**

Weighting strategies were used to take into account non-response in the survey so that the results were truly representative of the household population in England aged 16 years and over. Full details of these weighting strategies are detailed Chapter 13 of the main APSM report [23], but a paraphrased summary is provided below for easier reference.

Weighting of data from first phase of the survey took place in three steps:

*First* sample weights were applied to take into account the different probabilities of selecting respondents in households of different sizes;

*Second*, to reduce non-response bias at household level, a household level weight was calculated from a logistic regression model using interviewer observation and area-level variables collected from data from the 2001 Census;

*Finally*, weights were applied using calibration weighting techniques based on age, sex and region so that data represented the structure of the national population of England, and to take account of differential non-response between regions and age-by-sex groups.

Weighting of data from the second phase of the survey, accounts for the fact that :

- Participants eligible for phase two interviews were not selected with equal probability (e.g. those with higher scores obtained at phase two are likely to have had a higher probability of having been selected, and those with co-morbid mental disorders were selected with higher probabilities than those with one disorder);
- A proportion of participants selected for phase two interview declined to take part predisposing to non-response bias. A non-response adjustment to the weights was thus made.

Importantly, these phase two weights were designed to generate condition-specific datasets that were representative of the population ‘eligible’ for phase two specific conditions, e.g. for psychosis, the phase two weighted dataset represents those who had screened positive for “probable psychosis” at phase one interview.

**Supplementary note 2 – Identification of participants with psychosis**

Details on these procedures have been reported extensively in the original APMS report [23]. A paraphrased summary is provided below for ease of reference:

People with probable psychotic disorder were identified using data from the first phase of the survey questionnaire (*n*=7403), if they met one of the following criteria:

1. currently taking antipsychotic medication (either oral or long-acting depot injections);
2. reporting an in-patient stay for a mental or emotional problem in the previous three months, or an admission to a hospital or ward specialising in mental health at any time;
3. a positive response to question 5a that covers auditory hallucinations on the Psychosis Screening Questionnaire (PSQ) [43].
4. a self-reported diagnosis of a psychotic disorder or symptoms suggestive of it.

Of these, 313 (4%) met at least one of these screening criteria, making them eligible for a phase two clinical assessment for definitive psychosis using the Schedule for Clinical Assessment in Neuropsychiatry version 2.1 (SCAN) [25]. The latter is a semi-structured interview schedule that provides ICD-10 diagnoses of a psychotic disorder proper. Of these, 190 (61%) respondents provided a productive interview.

For our analysis, we assigned participants with either probable or definitive psychotic disorder in the previous year to a single “psychosis group”.

**STable 1.**

Summary of psychiatric treatment and mental health service utilisation reported by people with severe mental illness compared with people with common mental disorders

| *Variable* | SMI^a^  (*n*=40) | | CMDs^a^  (*n*=1248) | | | *P*^c^ |
| --- | --- | --- | --- | --- | --- | --- |
|  | *n* | (%) |  | *n* | (%) |  |
| *Current psychiatric treatment* |  |  |  |  |  |  |
| Taking psychotropic drugs^d^  Any psychotropics  Antipsychotics  Antidepressants  Anxiolytics  Hypnotics | 29  23  14  7  2 | (72.9)  (56.5)  (35.8)  (18.4)  (4.8) |  | 259  12  221  38  21 | (17.9)  (1.0)  (15.1)  (2.60)  (1.6) | < 0.001  < 0.001  0.012  < 0.001  0.263 |
| Undergoing psychotherapy^e^ | 15 | (40.2) |  | 116 | (8.8) | < 0.001 |
| Receiving both drugs and psychotherapy | 12 | (34.0) |  | 58 | (4.7) | < 0.001 |
| *Mental health service utilisation* |  |  |  |  |  |  |
| Seen psychiatrist^f^ | 12 | (32.0) |  | 27 | (2.7) | < 0.001 |
| Seen psychologist^f^ | 5 | (12.4) |  | 28 | (2.1) | < 0.001 |
| Seen community psychiatric nurse^f^ | 15 | (34.2) |  | 39 | (2.5) | < 0.001 |
| Attended out-patient psychiatric clinic^g^ | 9 | (27.1) |  | 41 | (3.4) | < 0.001 |
| Admitted to in-patient psychiatric facility^g^ | 3 | (5.8) |  | 5 | (0.3) | < 0.001 |
| Ever admitted to in-patient psychiatric facility | 25 | (62.8) |  | 75 | (5.1) | < 0.001 |
| Spoke to GP about mental health problem^f^ | 28 | (71.4) |  | 512 | (41.3) | < 0.001 |

^a^ Percentages are weighted to account for complex survey design

^b^ CMDs as reference group

^c^ Using Pearson’s χ² with Rao and Scott second order correction for survey data analysis

^d^ A complete list of psychotropic drugs is provided in McManus et al. (2007)

^e^ Any form of psychotherapy, psychoanalysis or counseling in individual or group format

^f^ In past year

^g^ In past quarter

*CI*: confidence interval; *CMD*: common mental disorder; *GP*: general practitioner; *OR*: odds ratio; *SMI*: severe mental illness

**STable 2.**

Physical conditions reported by people with severe mental illness compared with the general population: results of multivariable logistic regression models

| ***Variable*** | Adjusted OR^b^ (95% CI) |
| --- | --- |
|  |  |
| *Lifetime physical conditions*^a^ |  |
| None | – |
| 1 | 1.86 (0.55-6.79) |
| 2–3 | 4.30 (1.24-14.9)* |
| ≥4 | 6.11 (1.37-27.26)* |
| *Past-year physical conditions* |  |
| None | – |
| 1 | 3.30 (1.01-10.93)* |
| 2-3 | 3.57 (1.03-12.42)* |
| ≥4 | 7.38 (1.66-32.75)* |
| *Physical health service utilisation^c^* |  |
| Spoke to GP in past year^d^ | 2.42 (1.07-5.51)* |
| Attended out-patient clinic^e^ | 1.29 (0.56-3.00) |
| Admitted to hospital^e^ | 0.54 (0.07-0.83)* |

^a^Self-reported lifetime prevalence since the age of 16

^b^Adjusted for age, body mass index, gender, ethnicity, education, employment, smoking,

alcohol consumption and drug misuse

^d^Additionally adjusted for number of physical disorders

^d^In person or by telephone regarding a physical health problem

^e^For physical health problem only in previous quarter

**p* value < 0.05

*CI*: confidence interval; *OR*: odds ratio

*CI*: confidence interval; *CMD*: common mental disorder; *GP*: general practitioner; *OR*: odds ratio

^a^Self-reported lifetime condition since the age of 16

^b^Percentages are weighted to account for complex survey design

^c^Adjusted for age, body mass index, gender, ethnicity, education, employment, smoking, alcohol consumption and drug misuse
